# Intratesticular injection followed by electroporation allows gene transfer in caprine spermatogenic cells

**DOI:** 10.1038/s41598-018-21558-9

**Published:** 2018-02-16

**Authors:** R. Kumar Pramod, Abhijit Mitra

**Affiliations:** 1 Genome Analysis Laboratory, Animal Genetics Division, ICAR-Indian Veterinary Research Institute, Izatnagar, Bareilly, 243122 UP India; 2 Present Address: ICAR-National Research Centre on Mithun, Medziphema, Dimapur, Nagaland India; 3 Present Address: AgriGenome Labs Pvt. Ltd., Kakkanad, Cochin, Kerala India

## Abstract

The production of transgenic livestock is constrained due to the limited success of currently available methods for transgenesis. Testis mediated gene transfer (TMGT) is an emerging method that shows a high success rate in generating transgenic mice. In this study, we report a newly developed protocol for electroporation-aided TMGT to produce a transgenic goat. The injectable volume and concentration of the transgene were first standardized, and then electroporation conditions were optimized *in vitro*. *In vivo* experiments were performed by injecting a transgenic construct (*pIRES2-EGFP*; enhanced green fluorescent protein) into the testicular interstitium followed by electroporation. Immunohistochemistry, quantitative real-time PCR (qPCR) and western blotting analyses confirmed the successful transfer of the transgene into seminiferous tubules and testicular cells. Furthermore, chromosomal integration of the transgene and its expression in sperm were evaluated d60 and d120 post-electroporation. Our protocol neither altered the seminal characteristics nor the fertilization capacity of the sperm cells. *In vitro* fertilization using transgenic sperm generated fluorescent embryos. Finally, natural mating of a pre-founder buck produced a transgenic baby goat. The present study demonstrates the first successful report of an electroporation-aided TMGT method for gene transfer in goats.

## Introduction

The production of transgenic animals has predominantly been attempted by manipulating embryos, using a variety of techniques such as pronuclear microinjection, embryonic stem cell-mediated methods, and viral-mediated transfection^1–3^. Most of these methods, particularly pronuclear microinjection, are afflicted by poor efficiency, highly specialized laboratory techniques and skilled early embryonic manipulation^1,2,4,5^. During the last decade, the targeting of male germ cells has emerged as an alternative for transgenic animal production^6,7^. Generally, two strategies for gene transfer to male germ cells are employed; (1) sperm-mediated gene transfer (SMGT), and (2) testis-mediated gene transfer (TMGT). SMGT includes the direct transfer of genes into sperm cells, whereas TMGT involves *in vivo* introduction of foreign DNA into testicular germ cells to produce transgenic sperm cells. While SMGT appears to be a straightforward method, it suffers from poor repeatability and interspecies/intraspecies success variability^7^. On the other hand, the TMGT method, which involves surgical steps, presents a risk of infection and/or impotency if appropriate precautions are not taken. Nevertheless, TMGT allows for mass gene transfer by natural mating, exempting the use of cumbersome procedures such as *in vitro* fertilization and embryo transfer^8^. Furthermore, TMGT ensures a greater probability of stable integration of transgenes into the genome of the host animal^8,9^.

Several strategies, including viral, non-viral, physical and chemical methods, are employed in TMGT^8,10^. Owing to its higher efficiency, virus-mediated TMGT has become a preferred method, but it is limited by the harmful effects of inflammation^11^. Among non-viral methods, both lipofection-aided and electroporation-aided TMGT are considered to be easier and safer methods^8,12^. Further, *in vivo* electroporation is a safe method for gene transfer, as it does not cause adverse effects on testicular integrity or the fertilizing ability of spermatozoa^13,14^. Transgenic laboratory animals have been produced successfully using electroporation-aided TMGT^8,15^. However, the success rate of electroporation-aided TMGT depends on the sites of transgene injection, namely, the lumen of seminiferous tubules, rete testis or interstitium of the testis^8,9,15^. The direct injection of transgenes into the interstitium of the testis has shown a higher success rate than injection into the seminiferous tubules or rete testis, because the former site better facilitates the access of the transgene to undifferentiated spermatogonial germ cells^8^.

Available literature suggests an immense potential for electroporation-aided TMGT to produce transgenic laboratory animals^8,15^. Nevertheless, the enormous variation between species in the size, shape, and structures of testes necessitates the development of species-specific protocols. The goat is an ideal livestock species amenable to transgenesis. One specific application for goats is as mammary gland bioreactors. In addition, goats have smaller body size, a shorter gestation period, a higher prolificacy, and a relatively high content of protein in their milk^16,17^. The objective of the present study is to develop a protocol for electroporation-aided TMGT in goats.

## Results

### *In vitro* gene transfer and transgene expression in testis

First, we optimized the volume and concentration of the transgenic construct, linearized *pIRES2-EGFP* plasmid, by *in vitro* transfection of goat testis.

#### Injection volume

To optimize the injection volume, different volumes of phosphate-buffered saline (PBS) were injected into the testes. It was observed that the testis of pre-pubertal and adult bucks could accommodate a maximum of 1.0 and 1.5 ml of PBS, respectively. An increase in volume beyond the optimized level caused an apparent swelling of the testis.

#### DNA concentration

**Figure 1:**
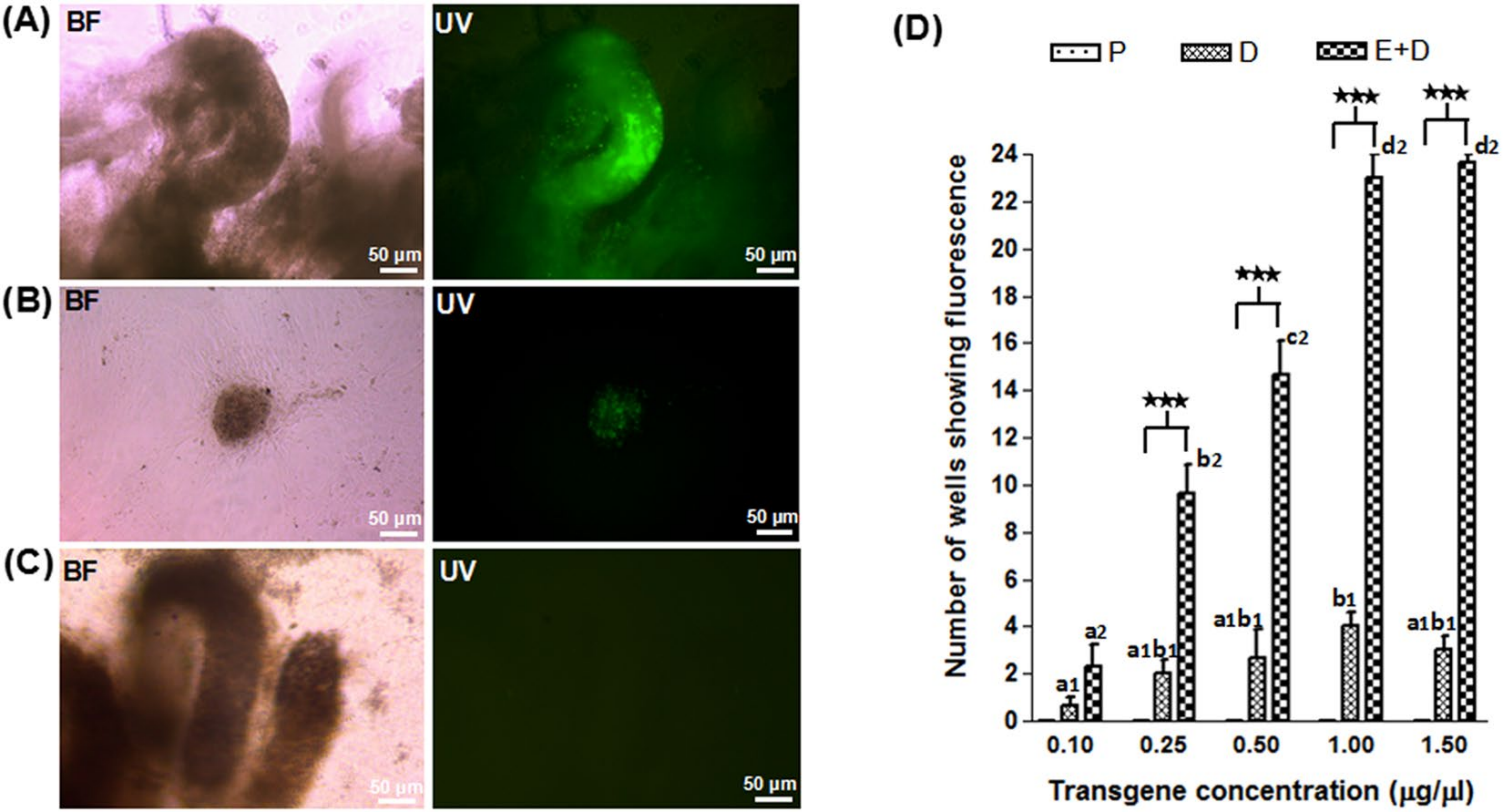
*EGFP* expression in the goat testes transfected *in vitro*. *EGFP* expression was observed as green fluorescence in (**A**) cultured seminiferous tubules and (**B**) spermatogonial stem cell (SSC) colonies; (**C**) An absence of fluorescence was observed in cultured seminiferous tubules from the testis injected with only PBS (negative control). BF: Bright field, UV: Under UV light. Scale bar, 50 μm; (**D**) Graphical representation of transfection efficiency using different plasmid concentrations (0.1, 0.25, 0.5, 1.0 and 1.5 µg) in goat testis. After *in vitro* transfection, seminiferous tubules were isolated and cultured in 24-well plates. The X-axis denotes plasmid concentration and the Y-axis represents the number of wells showing *EGFP* expression in seminiferous tubules. P: PBS injection only, D: injection of plasmid DNA without electroporation and E + D: injection of plasmid DNA followed by electroporation. Data represent the mean ± s.e.m. for n = 3. Letters (a–d) above the bars indicate that these groups differ significantly. In the E + D, *P* < 0.05 (between 0.1 µg and 0.25 µg), *P* < 0.05 (between 0.25 µg and 0.5 µg) and *P* < 0.01 (between 0.5 µg and 1.0 µg). Between the D and E + D groups, ***P < 0.001.

Under the optimum electroporation conditions, injecting the linearized plasmid at a concentration of 1 µg/µl resulted in a maximum *EGFP* expression in the seminiferous tubules (Fig. 1A) and spermatogonial stem cell (SSC) colonies (Fig. 1B). The EGFP protein expression was visible (green fluorescence) as early as day 3 (d3), and it lasted for more than three weeks in electroporated samples, suggesting non-episomal expression. In the PBS control group, no expression of *EGFP* was observed in the seminiferous tubules (Fig. 1C). In the absence of electroporation, there was no improvement in the efficiency of expression with increased plasmid concentration. However, the efficiency of expression was improved significantly when the plasmid concentration was increased from 0.1 to 1.0 µg/µl (Fig. 1D). However, the expression did not change significantly when the plasmid concentration was further increased to 1.5 µg/µl. For all the plasmid concentrations except 0.1 µg/µl, the non-electroporated samples showed ~five times lower (*P* < *0.001*) efficiency in expressing *EGFP* (Supplementary Table S1).

### Expression of *EGFP* in pre-pubertal goat testes after *in vivo* gene transfer

**Figure 2:**
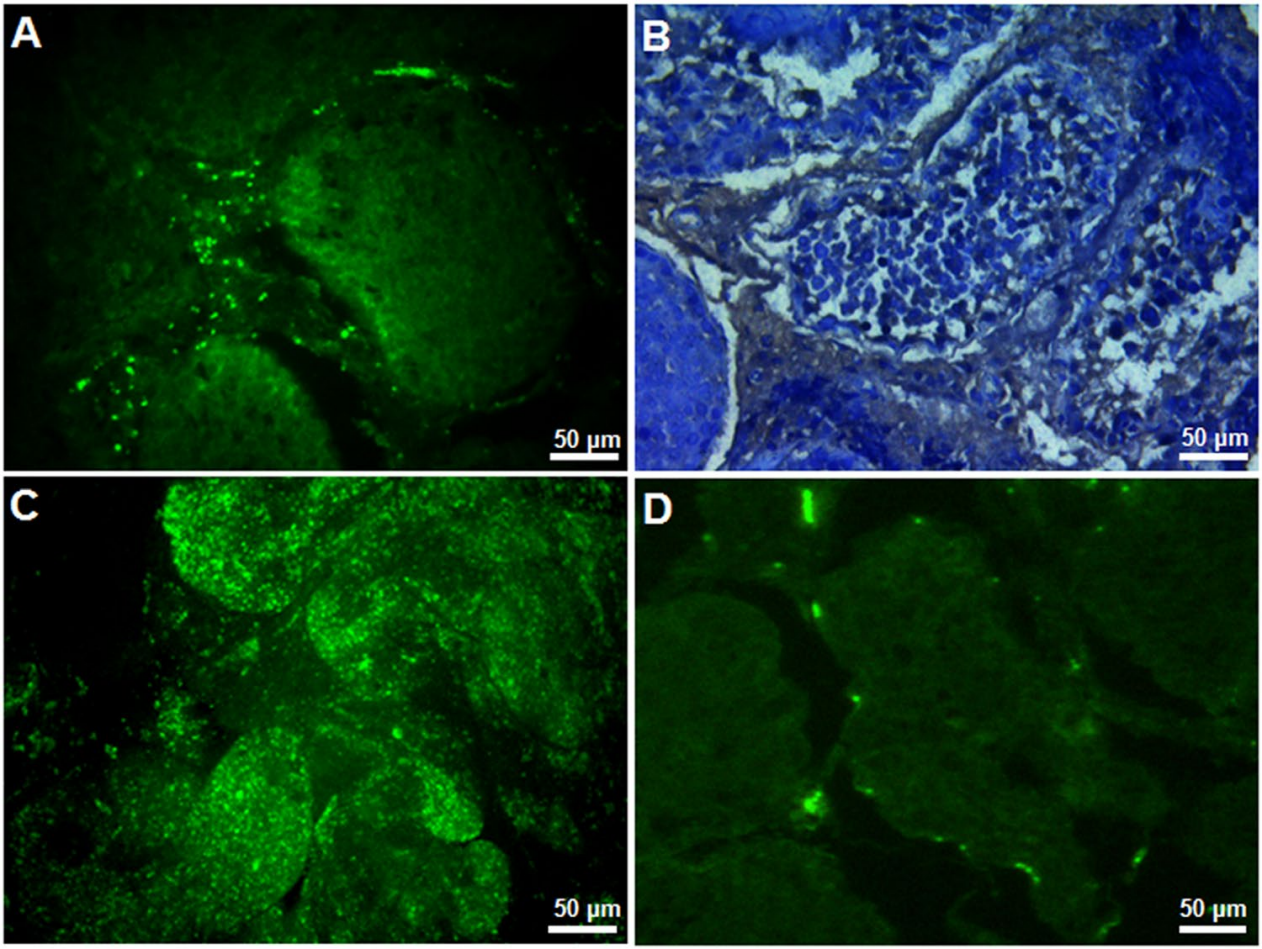
Expression of *EGFP* in testicular cells on d21 after *in vivo* gene transfer. (**A**) Direct fluorescence microscopy of a testis cross-section showing green fluorescence in testicular cells; (**B**) IHC revealed the localization *EGFP* protein (in brown) in almost all the testicular cells including spermatogonial cells; (**C**) clusters of green fluorescent cells in seminiferous tubules; (**D**) IHC analysis of testis showing the location of SSCs in the basement membrane of seminiferous tubules. The α6 integrin antibody was used as a specific marker for SSCs. Scale bar, 50 μm.

**Figure 3:**
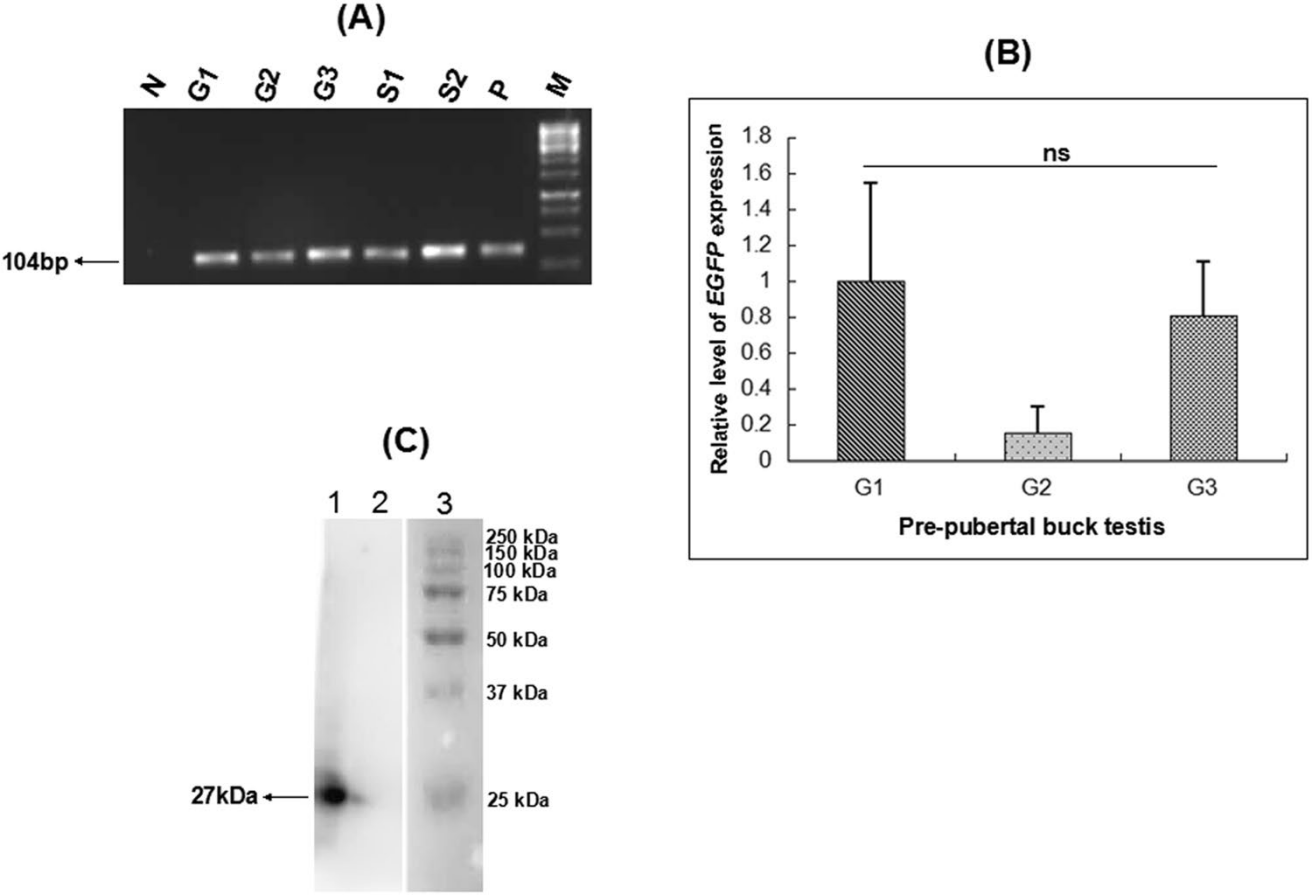
*EGFP* expression in the testis of pre-pubertal bucks on d21 after *in vivo* gene transfer. (**A**) Gel electrophoresis of quantitative real-time PCR products from gene transferred testis revealed an *EGFP*-specific band of 104 bp. The top of the gel with the wells was cropped; N: no template control (NTC), G1-G3: testicular tissue from pre-pubertal goats with gene transferred testis, S1 & S2: cultured seminiferous tubules from *in vitro* transfected testes, P: positive control (pIRES2-*EGFP* plasmid), M: 100 bp molecular marker; (**B**) graphical representation of the relative expression of *EGFP* mRNA in testicular tissues from pre-pubertal bucks at d21 post-gene transfer. G1, G2 & G3 are pre-pubertal bucks #1, #2 & #3, respectively. Data represent the mean ± s.e.m. for n = 3. Here, ‘ns’ means statistically not significant; (**C**) an approx. 27 kDa protein band was observed by western blotting analysis, using total protein from testicular tissue of an *in vivo* electroporated goat. The marker lane was cut separately before incubating the membrane with the primary antibody. The wells are labeled 1: gene transferred testis of a pre-pubertal goat, 2: testis of a non-gene transferred goat, 3: protein marker (#1610373, Bio-Rad).

On d21 post-electroporation, microscopic examination of testes revealed EGFP expression in spermatogenic cells, Sertoli cells and other interstitial cells (Fig. 2A). Immunohistochemical (IHC) analysis of tissue sections of transfected testes showed localization of EGFP protein (brown color) in spermatogonial cells, adjacent to the basement membrane of seminiferous tubules (Fig. 2B). Seminiferous tubules showed clusters of green fluorescent cells (Fig. 2C). The *EGFP* expression was further confirmed by quantitative real-time PCR (qPCR) (Fig. 3A and B). Western blot analysis of transfected testes showed a 27 kDa EFGP protein (Fig. 3C).

### Effects of electroporation-mediated gene transfer on semen fertility parameters

**Figure 4:**
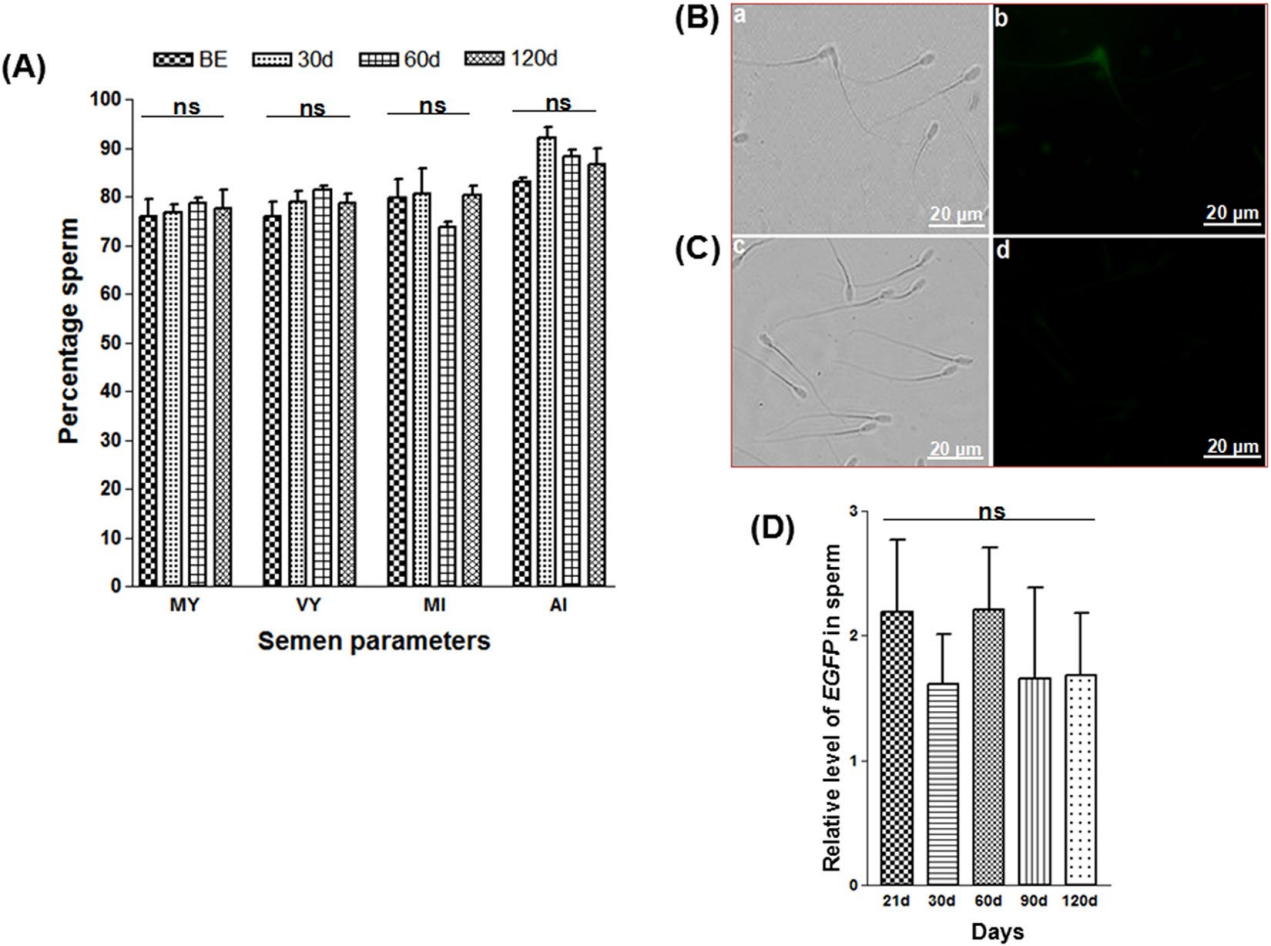
Assessment of semen samples obtained from bucks with transfected testis. (**A**) The semen parameters showed no variation before and after gene transfer. Semen from three *in vivo* gene transferred bucks (n = 3) were assessed at different intervals. BE: before electroporation, MY: progressive motility, VY: viability, MI: membrane integrity, AI: acrosome integrity. Data represent the mean ± s.e.m.; (**B**) *EGFP* expression in the sperm from a gene transferred buck; (**C**) no expression of *EGFP* was observed in sperm from a wild buck. (a & c): Bright-field, (b & d): under UV light. Scale bar, 20 μm; (**D**) The graph shows qPCR analysis of sperm from *in vivo* gene transferred bucks at different intervals indicating the genomic integration of transgene. Data represent the mean ± s.e.m. for n = 3. Here, ‘ns’ means statistically not significant.

The vital semen parameters, namely, progressive motility, viability, membrane integrity and acrosome integrity, did not vary significantly among the semen samples collected from the experimental bucks before and after electroporation (Fig. 4A). This result suggests the absence of any detrimental effects from electroporation-mediated gene transfer on the sperm quality.

### Integration and expression of the transgene in the sperm

Microscopic examinations of sperms on d60 showed a limited (0.83%) number of cells containing green fluorescent protein (Fig. 4B,C and Supplementary Table S2). However, we failed to observe *EGFP* transcripts in the semen by reverse transcription PCR (RT-PCR) (data not shown). qPCR analysis of semen samples from all three bucks revealed the presence of the *EGFP* gene until d120 post-electroporation, further confirming the chromosomal integration of the transgene into the sperm (Fig. 4D).

### Transgenic embryo production using semen from an *in vivo* gene transferred buck

**Figure 5:**
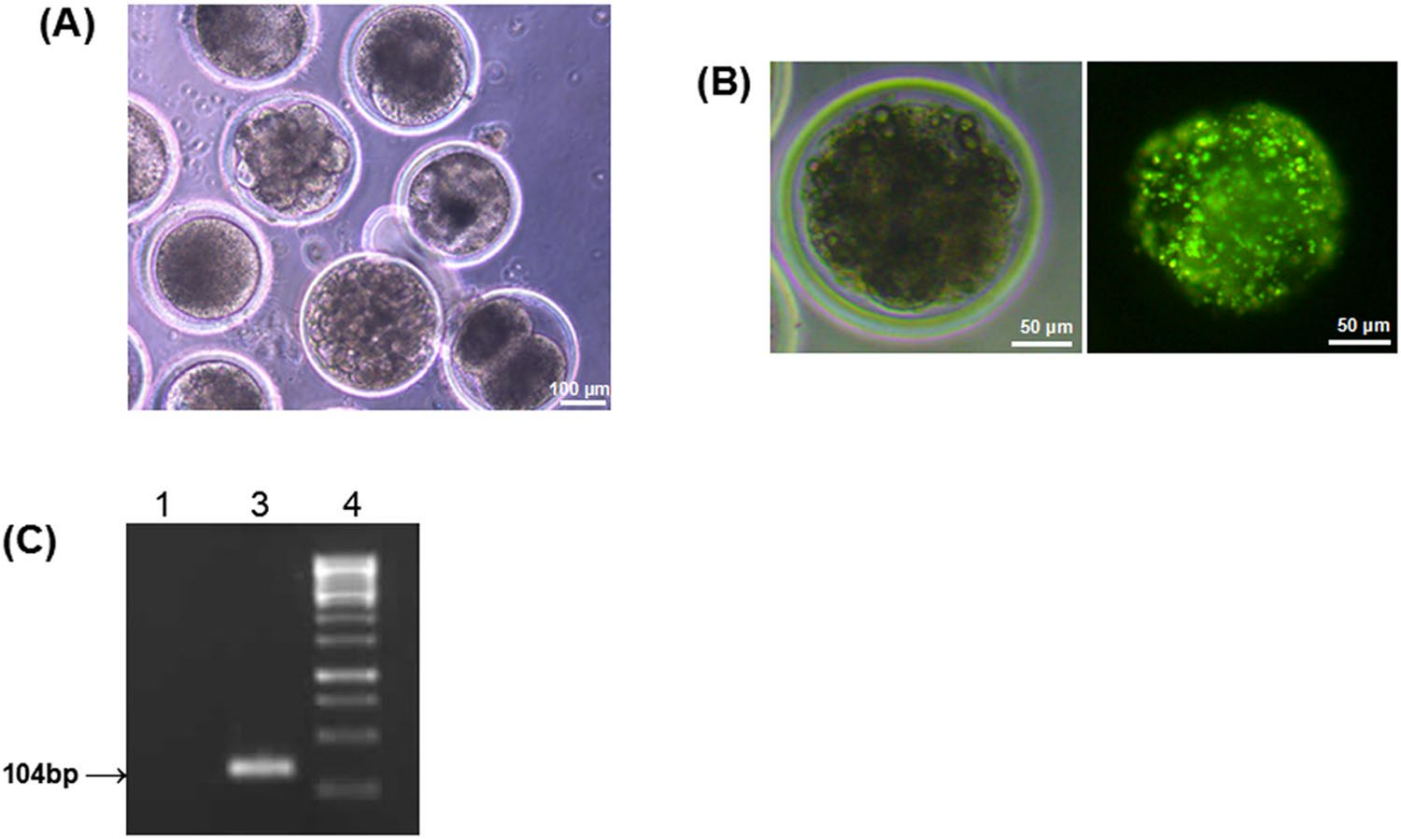
Detection of *EGFP* expression in embryos produced *in vitro* using semen from *in vivo* gene transferred bucks. (**A**) *In vitro* produced embryos (pooled). Scale bar, 100 μm; (**B**) *EGFP* expression in the embryo (a) under bright-field and (b) UV light. Scale bar, 50 μm; (**C**) RT-PCR analysis revealed expression of *EGFP* mRNA in the fluorescent embryo. 1; normal embryo, 2; *EGFP-*expressing embryo, 3; 100 bp molecular marker. The gel picture was cropped, and the full-length gel is presented in Supplementary Figure 5C.

The effects of electroporation and foreign DNA on the fertilizing ability of the sperm was assessed using an *in vitro* fertilization (IVF) assay. There was no significant difference in the cleavage rate between the IVF embryos obtained using the semen from transfected (22.00 ± 1.30) and non-transfected (23.40 ± 1.07) bucks (Fig. 5A). Out of 110 embryos analyzed, three embryos (2.72%) showed transgenic expression of green fluorescence (Fig. 5B). RT-PCR analysis of these fluorescent embryos also confirmed the presence of *EGFP* mRNA (Fig. 5C).

### Production of a transgenic kid

**Figure 6:**
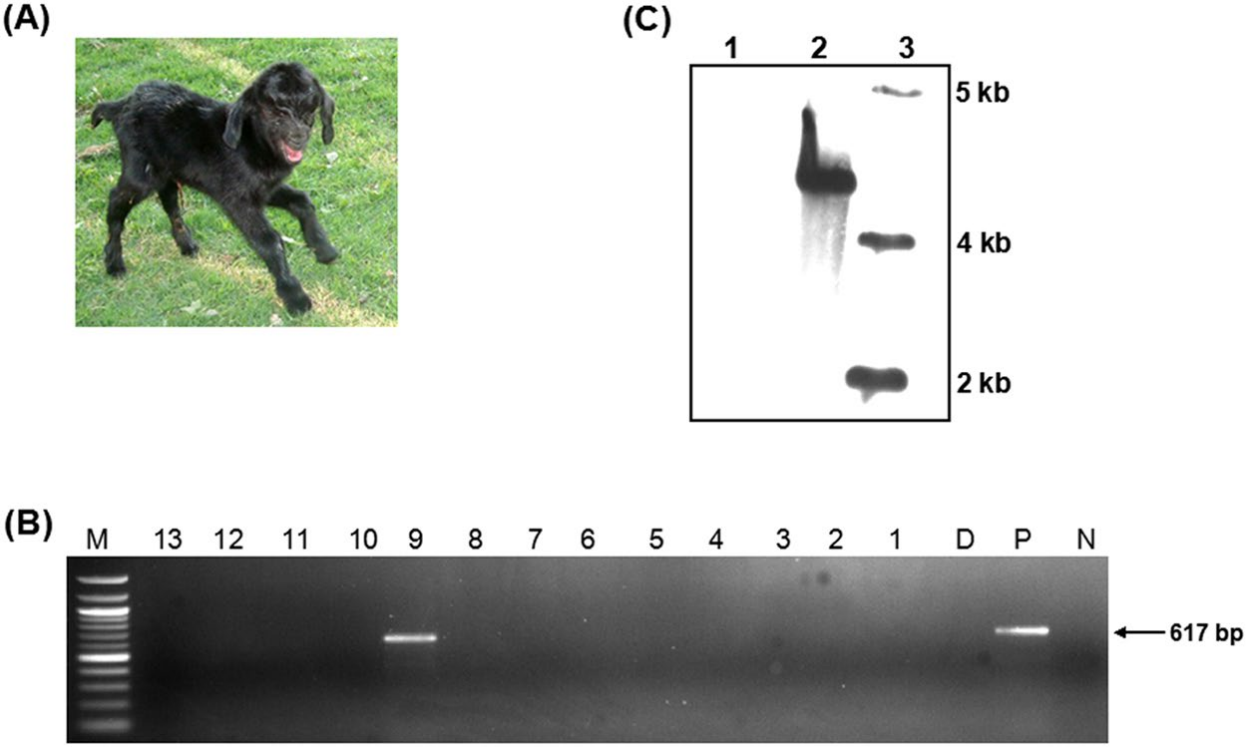
Generation of a transgenic kid from an *in vivo* gene transferred buck. A total of nine matings of three bucks carrying electroporated testis resulted in the birth of 13 kids. (**A**) Representative photograph of a transgenic baby goat. The kid showed no *EGFP* expression; (**B**) PCR analysis revealed the presence of the *EGFP* gene in the blood genomic DNA of the kid, indicating the integration of plasmid into the germline cells of the *in vivo* gene transferred buck. Lane 13–10 & lane 8–1: kids without transgene in their genome, 9: kid carrying the transgene, D: normal doe, P: positive control (pIRES2-*EGFP* plasmid), N: no-template control (NTC), M: 100 bp DNA ladder. The top of the gel containing the wells was cropped; (**C**) Southern blot analysis of genomic DNA from the transgenic kid, 1: genomic DNA from a wild goat, 2: DNA from the kid carrying the transgene, 3: DNA marker. The blot image was cropped, and the full-length blot is presented in Supplementary Figure 6C.

The optimized procedure for transgenic baby goat production was assessed. A total of nine matings of three pre-founder bucks resulted in the birth of 13 kids (Supplementary Table S3). PCR analysis revealed the presence of pIRES2-*EGFP* in one kid, indicating that the transgene was integrated into the spermatogonial cells of the pre-founder male (Fig. 6A and B). This result was further confirmed using Southern blot analysis (Fig. 6C). However, neither fluorescence nor the *EGFP* transcript were detected in the blood or skin of the transgenic kid.

## Discussion

Transgenic livestock animals have the potential to act as bioreactors for generating large quantities of biologically active proteins^18,19^. Nevertheless, most of the traditional methods for transgenic livestock production, particularly pronuclear microinjection, are constrained by limited success rates^2,20^. The lentiviral-mediated gene transfer method shows a comparatively higher efficiency but suffers from insertional mutagenesis^21–23^ and limitations in transgene-carrying capacity^24,25^. Recently, electroporation-mediated TMGT has emerged as an alternative method for transgenic animal production with a high success rate^8^, but it is restricted to laboratory animal species such as mice. In this study, we report for the first time a successful transgenesis method using electroporation-aided TMGT in goat.

Previous studies dealing with TMGT in laboratory animals investigated different concentrations and volumes of exogenous DNA with varying success rates^8,14,15^. In the present study, TMGT was employed for *in vitro* gene transfection of testes. First, we optimized the injection volume and the concentration of the transgenic vector. The results showed that the testes of a pre-pubertal and adult buck can accommodate injection volumes as high as 1.0 and 1.5 ml, respectively. The variation in the injection volume is possibly due to the change in testicular size with age and the onset of puberty^26–28^ (Supplementary Table S4). Using trypan blue solution as a marker dye^9,14^, we further confirmed that injection volumes of 1.0 and 1.5 ml are sufficient to cover the entire testicular area (Supplementary Figure S1). The results of *in vitro* transfection experiments further demonstrate that 1 µg/µl is an optimum plasmid concentration for gene transfer.

*In vivo* electroporation-mediated transfection studies of large mammals are very limited compared to those of laboratory animals. In human, a transdermal voltage of at least 50 V is necessary to cause a significant molecular transport across the skin, regardless of the fluorescent tracer used^29^. A range of 25 to 50 mV was shown to be the most efficient condition for electroporation-mediated transfection of SSCs in neonatal bovine testicular tissue *in vitro*^30^. However, in laboratory animals, the most favorable voltage ranges from 30 to 50 V, which guarantees minimal adverse effects on testicular integrity, a normal sperm quality, the maintenance of offspring production ability and sufficient transfection efficacy^8,9,14^. An increase in transfection efficiency by raising the voltage is accompanied by the adverse effect of testicular shrinking^31^. Further, we observed visible discomfort in the animal when we attempted a trial with a higher voltage (data not shown). Accordingly, in compliance with strict bio-ethical concerns and in the absence of any cited literature using a voltage higher than 50 mV, we employed the previously reported electroporation conditions (50 V and 50 ms) from our laboratory^32^.

It is now known that the site of injection greatly influences the success of TMGT. Previous studies have suggested that transgenic constructs can be injected into either the lumen of seminiferous tubules^9^, rete testis^13^ or in the interstitium^8^. Later, nonsurgical *in vivo* electroporation of testis was reported to be successful for gene transfer through the testicular interstitium^33^. In another study, researchers demonstrated the expression of *GFP* in the seminiferous tubules for more than two months when the transgenic construct was directly injected into the seminiferous tubules of mice, but they failed to produce a transgenic pup^9^. However, in the same year, another group reported the production of transgenic mice by intracytoplasmic sperm injection (ICSI) using fluorescent spermatozoa that were derived from the injection of a transgenic construct through the rete testis^15^. TMGT via direct injection into the rete testis in hamster, a non-murine species, also revealed the expression of the transgene in epididymal sperms on d60 post-electroporation^14^. In the current study, we injected the vector into the testicular interstitium and demonstrated the expression of *EGFP* in the seminiferous tubules, even on d21 after *in vivo* gene transfer, indicating a non-episomal expression of the transgene. IHC analysis further showed *EGFP* expression in the spermatogonial cells as well as other interstitial cells of the testis. The delivery of the transgene into the testicular interstitium *in situ* might have allowed for direct access to the undifferentiated spermatogonial cells, due to their location in the basement membrane of the seminiferous tubules^8^.

In the present study, we ascertained that neither electroporation nor the presence of foreign DNA affected the spermatogenic process and/or deteriorated the semen quality or the fertilizing ability of sperm. Except in hamster^14^, the effects of electroporation-mediated TMGT on semen quality have scarcely been reported. That study reported no adverse effects on the motility and viability of hamster sperm. The results of our study strengthen the previous findings, and we did not observe distinguishable changes in the vital parameters of semen from the experimental (i.e., with the electroporated testes) and control bucks. Moreover, embryos obtained by *in vitro* fertilization with the semen from experimental and control bucks showed similar cleavage rates. These results agree with literature^14^ stating that electroporation-mediated TMGT neither affects sperm function nor their fertilizing ability and subsequent embryonic development.

Our study assessed the integration of the transgene into the spermatogenic cells of pre-founder males. The most convincing direct evidence of genomic integration is the presence of fluorescent spermatozoa when examined under a fluorescence microscope. In a previous study performing TMGT in mice, the presence of clusters of fluorescent spermatozoa in the seminiferous tubules showed the expression of yellow fluorescent protein (*YFP*)^15^. The authors suggested that the absence of *GFP* expression in sperm was either due to potential toxicity from the GFP or a very low rate of chromosomal integration. In TMGT using hamsters, *YFP* fluorescence was detected in the mid-piece of approximately 10% of epididymal sperm^14^. Subsequently, Chandrashekran and co-workers showed *GFP* expression in porcine sperm by direct transfection with a pseudotyped lentiviral vector^34^. Other researchers observed fluorescent pig spermatozoa using *GFP*^35^ and Venus (yellow shifted variant of *EGFP*)^36^ constructs. The absence of Venus mRNA in the sperm, however, suggested the presence of Venus protein that had already been translated in pro-spermatogonial stages^36^. Using the CMV promoter, researchers demonstrated *GFP* expression in mice spermatogonia and round spermatids, but not in the spermatozoa^37^. We observed minimal *EGFP* expression under the CMV promoter in caprine spermatozoa, but we failed to observe *EGFP* mRNA in their semen. The green fluorescence in spermatozoa observed in the present study and in other work^36^ is likely due to the presence of *EGFP* protein that had already been translated in pre-prospermatogonial or spermatogonial stages. Contrary to earlier observations that mature spermatozoa lose most of their cytoplasm and become transcriptionally dormant^38,39^, recent studies have suggested the presence of remnant mRNAs in the mature spermatozoa of several mammals^40,41^.

Although our study demonstrated *EGFP* expression in less than 1% of sperm, the percentages of fluorescent embryos and transgenic kids were 2.72% and 7.69%, respectively. Further, RT-PCR and western blot analysis confirmed *EGFP* expression in different parts of the testis, and IHC demonstrated EGFP localization in spermatogonial cells near the basement membrane of seminiferous tubules. Finally, qPCR analysis confirmed the presence of the *EGFP* gene until d120 after electroporation. In goats, the spermatogenic cycle is approximately 47.7 days^42^. Therefore, the presence of *EGFP* in the sperm, even up to 120 d after electroporation, confirms the chromosomal integration of *EGFP* into SSCs and thus agrees with the literature^9,14^.

Based on the results of this study, for the first time we have demonstrated the production of fluorescent IVF goat embryos using electroporation-mediated TMGT. The cleavage rates of embryos obtained after IVF using semen from either experimental or control bucks were comparable in this study. In our earlier report, we observed 4.31% fluorescent embryos while using SMGT^43^. Previously, another group also produced transgenic embryos using intra-cytoplasmic sperm injection (ICSI) of fluorescent sperm in mice^15^. The smaller number of fluorescent embryos produced in this study might be due to several reasons. First, there might be fewer sperms carrying the transgene than non-transgenic ones. Second, oocytes may have been preferentially fertilized with non-transgenic sperm, since transgenic sperm may be less competitive in reaching the oocytes or in penetrating the egg coats^44,45^. The interaction of exogenous DNA with sperm cells is also known to activate endogenous nucleases, which in turn might degrade the foreign DNA and/or sperm chromosomal DNA^46^. However, such a possibility seems to be remote in TMGT, as the transgene integration should have actually taken place in the progenitors of the sperm cells, i.e., SSCs.

Electroporation-aided TMGT has been reported with low^9,47^ to high success^8^ rates in laboratory animals. In the present study, one transgenic kid was born using electroporation-aided TMGT. The ability of the pre-founder buck to sire a transgenic kid after d60 post-electroporation suggested the integration of the transgene into the spermatogonial cells. However, we failed to detect either *in vivo* fluorescence or *EGFP* transcripts in the kid. Other studies have also reported the absence of *EGFP* expression in transgenic offspring due to either a low copy number of the transgene or mosaicism^12,48^. Further, the expression of the reporter gene, as observed in pronuclear injection, might have been influenced by the highly likely event of random integration and concatemer formation by homologous recombination before integration into the host genome^49–51^. This limitation needs to be circumvented by using the recently developed gene editing technologies such as zinc finger nucleases (ZFNs)^52^, transcription activator-like effector nucleases (TALENs)^53^ or clustered regularly interspersed short palindromic repeat (CRISPR)/CRISPR-associated protein 9 (Cas9)^54^ to ensure targeted integration of transgenes. In the future, combination of any of the above technologies with TMGT could offer efficient gene transfer in large animals.

In conclusion, the present study is the first successful report of an electroporation-aided TMGT technique for *in vivo* transfection of spermatogenic cells in farm animals. The results suggest that the direct injection of a transgenic construct into the testicular interstitium followed by electroporation results in successful integration of a transgene into the genome of testicular cells. This method also allows for the integration of transgenes into spermatogonial cells without affecting the fertilizing ability of the spermatozoa. This electroporation-aided TMGT method, with the help of recent gene editing technologies, seems to be the most convenient and promising method for the generation of transgenic farm animals, including transgenic goats. Future studies are warranted to further optimize the procedure to recover more fluorescent spermatozoa to use for artificial insemination.

## Methods

### Experimental animals

The care and experimental use of animals were approved by the Institutional Animal Ethics Committee (IAEC) as well as the Institutional Biosafety Committee (IBC) of the Indian Veterinary Research Institute (IVRI), Uttar Pradesh, India. All the experiments were performed in accordance with the guidelines of the Review Committee on Genetic Manipulation (RCGM) under the Department of Biotechnology (DBT), India. Male goats maintained under identical management conditions at the experimental herd of our laboratory were included in the study. Animals were divided into two groups. The first group (n = 3) consisted of pre-pubertal male goats 3–4 of months of age and the second group (n = 3) included adult male goats approximately three years of age (bucks).

### *In vitro* transfection of goat testis using electroporation

The plasmid containing the *GFP* coding sequence (*pIRES2-EGFP*), a kind gift from Dr. Subeer Majumdar (NII, New Delhi) was used as a transgenic construct. The plasmid was isolated using an endotoxin-free quanta maxi kit (Advanced Microdevices Pvt. Ltd., India) and digested with the restriction enzyme *StuI* (Fermentas, Canada). The linearized plasmid was purified by ethanol precipitation and finally dissolved in sterilized PBS (pH 7.4).

Prior to the *in vivo* gene transfer experiment, a pilot study was conducted using pre-pubertal and adult goat testes collected from an abattoir in Bareilly, UP. Testes were immediately transported to the laboratory in PBS on the ice. In the laboratory, testes were washed with PBS containing 50 µg/ml Gentamicin (Sigma, USA). First, to optimize the injection volume of the transgenic construct, different volumes (0.25 to 2.0 ml) of PBS were injected into the interstitium of testes using a 27- G needle (BD Biosciences®, USA) attached to a tuberculin disposable syringe. The maximum volume of PBS that a testis can accommodate without any swelling was considered to be the optimum volume. The optimum volume was also confirmed by the injection of a 0.4% solution of trypan blue in buffered isotonic salt solution (pH 7.3) into the testicular interstitium (Supplementary Figure S1). To optimize the concentration (µg/µl) of the transgenic construct, the optimized volume of linearized plasmid DNA, at concentrations varying from 0.1 to1.5 µg/µl, was injected into ten different sites of the testicular interstitial space of pre-pubertal and adult goat testes (Supplementary Table S5). After each injection, the needle was removed very slowly to avoid any leakage due to the backflow of the injected solution.

Immediately after each injection, the testis was held between a pair of caliper-type electrodes (ECM830, BTX, USA, Item#45-0102) and square electric pulses were applied using electric pulse generator (ECM830, BTX, USA). A total of eight pulses (i.e., four pulses in one direction followed by another four pulses in the reverse direction) were applied. Each pulse was 50 V for 50 ms with an inter-pulse interval of 1s. After the electroporation, seminiferous tubules and SSCs were isolated from the testes and were cultured using a protocol developed in our laboratory^32,55^ (Supplementary materials and methods). To assess the efficiency of electroporation, one negative control (P: PBS only) and one electroporation control (D: only plasmid injection without electroporation) were included.

The cultures were maintained for more than four weeks. Starting from d3, SSCs were examined regularly for *EGFP* expression under a fluorescence microscope (Leica, Germany).

### *In vivo* gene transfer to the goat testis using electroporation

After the *in vitro* experiment, the left testis of each experimental animal was injected *in vivo* with the optimized volume and concentration of the aqueous solution of linearized plasmid DNA. A plasmid concentration of 1 µg/µl in PBS was used, at volumes of 1.0 ml 1.5 ml for pre-pubertal and adult goat testes, respectively. In the prepubertal goat, the right testis was kept completely untouched throughout the experiment as a control. However, in adult goats, the right testes were removed immediately after electroporation to prevent the dilution of transgenic sperms.

Before the electroporation procedure, regional anesthesia of the testicular area was achieved by epidural infiltration of lignocaine 2% (Xylocaine 2%, Astra Zeneca, UK). The animal was placed in lateral recumbency with the left hind limb up and away by exposing the surgical field. The scrotum and surrounding area were clipped and prepared aseptically for surgery. An incision was made on the caudoventral surface of the testis through the skin and tunica dartos. The testis was exposed in the scrotal sack and then fixed with the tip of fingers to avoid retraction during the injection. Injection and electroporation conditions were similar to those of the *in vitro* experiment. After electroporation, the testis was replaced. The non-injected testis was removed by separating the cremaster from the vascular testicular cord. Each of these structures was ligated by transfixation suturing. Finally, the cord was transected distal to the ligatures and the testis was removed. The muscle and skin layers were sutured leaving a small gap for the exudation (Supplementary Figure S2). An antiseptic dressing containing povidone-iodine was provided for healing. All the animals were housed in individual shelters and administered with broad-spectrum antibiotics.

### Detection of *EGFP* expression in the *in vivo* transfected testes

On d21 post-electroporation, testes (i.e., the left experimental and right control) from the prepubertal goats were surgically removed and were transported immediately to the laboratory on ice. In the laboratory, the epididymis was separated from the testis and testicular tissues were processed for further analysis.

A piece of each testis was cut into thin sections and the obtained testicular tissues were teased apart in PBS to separate the seminiferous tubules. The isolated seminiferous tubules were examined directly under a fluorescence microscope to assess the *EGFP* expression. A portion of the isolated seminiferous tubules was stored in RNAlater (Qiagen, Germany) for total RNA isolation, and another portion in T-PER reagent (Thermo Scientific, USA) for protein isolation, at −20 °C until further use. Histomorphology and IHC analysis of the testes were performed with tissue fixed in 4% paraformaldehyde (PFA) and sectioned at 5 µm thickness. IHC staining of fixed tissue samples was performed using anti- GFP antibody (Supplementary materials and methods).

Total RNA was extracted from the seminiferous tubules using TRI-reagent (Sigma, USA) as per the recommended protocol. One microgram of DNase-treated total RNA was reverse-transcribed using a cDNA synthesis kit (RevertAid H Minus First Strand cDNA Synthesis Kit, Fermentas, USA). The expression of *EGFP* was analyzed by qPCR on an Applied Biosystems 7500 Real-time PCR system (Supplementary materials and methods). β-actin (*ACTB*) was used as a reference gene for qPCR analysis. Total protein was extracted from the seminiferous tubules using T-PER reagent according to the manufacturer’s instructions. Total protein (30 μg per lane) was analyzed by western blotting according to standard procedures (Supplementary materials and methods).

### Evaluation of semen parameters

On d30, d60 and d120 after electroporation, semen samples were collected from the bucks (n = 3) using a sterilized artificial vagina (AV). Immediately after the semen collection, the tube containing semen was placed in a water bath (37 °C). Semen samples were analyzed to evaluate viability, motility, membrane integrity and acrosomal integrity. To assess the progressive motility, semen was diluted (1:10) with sodium citrate-glucose (SCG) buffer. One drop of the diluted semen was placed on a glass slide, and after placing a cover slip, it was immediately examined under the high power (40×) objective of a phase contrast microscope. At least 20 fields were examined and the average number of motile sperm cells (%) was determined. Eosin-Nigrosin based differential staining was used to determine the live/dead percentage of the sperms. A hypo-osmotic swelling test (HOST) was employed to assess the functional integrity of the sperm cell membranes^56^. The acrosomal integrity was determined by staining with fluorescein isothiocyanate coupled with peanut agglutinin (FITC-PSA)^57^.

### Estimation of transgene integration in the sperm

To assess the integration of the transgene into the sperm, semen samples from the transfected testes of all three bucks were collected at five different time intervals: d 21, 30, 60, 90 and 120 post-electroporation. Each 50 µl semen sample was washed four times with PBS and centrifuged for 5 min at 800 g to pellet the sperms. Total DNA from the pelleted sperm was isolated using a GeneipureID^TM^ DNA isolation kit (Genei, India) according to the manufacturer’s instructions. The presence of the *EGFP* gene in the sperm of transfected animals at different intervals was assessed using qPCR. β-actin was used as an internal control (Supplementary materials and methods). At d60, a portion of the semen samples was examined directly under a fluorescence microscope to detect *EGFP* expression in sperm. Approximately 200 sperms were examined from three representative microscopic fields for each semen sample and EGFP-positive sperms were counted. Further, RT-PCR analysis of sperm RNA was performed at d60 post-electroporation.

### Fertilizing ability of the sperms from the bucks with transfected testes

Semen samples from *in vivo* transfected testes were used for *in vitro* fertilization (IVF) to evaluate the effects of electroporation on the fertilizing ability of the sperm. Goat ovaries were obtained from the local abattoir and aspirated oocytes were matured, fertilized and cultured *in vitro* (Supplementary materials and methods). The cleaved embryos were assessed for *EGFP* expression under a fluorescence microscope. Fluorescent embryos were pooled, and total RNA was extracted using an RNeasy Mini Kit (Qiagen, Germany). Then, RT-PCR analysis was performed to assess the abundance of *EGFP* transcripts in the embryos.

### Transgenic screening of kids born out of natural mating of bucks with transfected testis

After d60 post transfection, bucks were allowed to mate naturally with the adult female goats (does) of similar age. Blood genomic DNA was isolated from all the kids born using phenol-chloroform extraction followed by ethanol precipitation^58^. The genotypes of all the kids were verified by PCR (Supplementary materials and methods) and Southern blot analysis (Supplementary materials and methods) to determine the presence of the *EGFP* reporter gene. *In vivo EGFP* fluorescence in the kids was assessed using a portable light source (excitation maximum = 488 nm; emission maximum = 507 nm). *EGFP* gene expression was also evaluated by RT-PCR analysis of blood and skin samples.

### Statistical analysis

The transfection efficiency of *in vitro* electroporation was evaluated using two-way ANOVA. One-way ANOVA was used to assess the effects of *in vivo* electroporation on the different semen parameters, the relative expression of *EGFP* mRNA in testis and the presence of the *EGFP* gene in sperm. The percentage data of the semen parameters were subjected to arcsine transformation before statistical analysis. A *P*-value < 0.05 was considered to be statistically significant.

## Electronic supplementary material

Supplementary information on caprine TMGT
