# Supplementary material for: Intratesticular injection followed by electroporation allows gene transfer in caprine spermatogenic cells
Source: Sci Rep. 2018 Feb 16;8:3169. doi: 10.1038/s41598-018-21558-9 (PMC5816633; doi:10.1038/s41598-018-21558-9)
Supplement: Supplementary file 1 — Supplementary information on caprine TMGT [file 41598_2018_21558_MOESM1_ESM.doc]

**Supplementary Information**

**Intratesticular injection followed by electroporation allows gene transfer in caprine spermatogenic cells**

R. Kumar Pramod and Abhijit Mitra

**Table S1. Number of wells showing *EGFP* expression.** Seminiferous tubules were isolated from the testes after *in vitro* transfection and cultured in 24-well plates.

| **Condition** | **P: PBS injection only** | **D: DNA injection without electroporation** | | | | | **E+D: DNA injection followed by electroporation** | | | | |
| --- | --- | --- | --- | --- | --- | --- | --- | --- | --- | --- | --- |
| **Plasmid (µg/µl)** | **0** | **0.1** | **0.25** | **0.50** | **1.0** | **1.5** | **0.1** | **0.25** | **0.50** | **1.0** | **1.5** |
| **Experiment 1** | 0 | 0 | 2 | 2 | 3 | 2 | 2 | 8 | 15 | 24 | 24 |
| **Experiment 2** | 0 | 1 | 3 | 5 | 5 | 4 | 1 | 12 | 12 | 21 | 24 |
| **Experiment 3** | 0 | 1 | 1 | 1 | 4 | 3 | 4 | 9 | 17 | 24 | 23 |
| **Mean ± s.e.m.** | 0 | 0.66±  0.33 | 2±  0.58 | 2.66±  1.20 | 4±  0.58 | 3±  0.58 | 2.33±  0.88 | 9.66±  1.20 | 14.6±  1.45 | 23±  1.0 | 23.6±  0.33 |
| **Efficiency %** | 0 | 2.77 | 8.33 | 11.11 | 16.67 | 12.50 | 9.72 | 40.27 | 61.11 | 95.83 | 98.61 |

Efficiency% = (number wells showing EGFP expression/ 24)*100

**Table S2. Number of fluorescent spermatozoa present in the semen samples of bucks with transfected testis on d60 post-electroporation.** Approximately 200 spermatozoa/field were examined and fluorescent sperms were counted. Out of three bucks, semen from two bucks showed green fluorescent sperm.

| Animal No | Field number | Number of fluorescent sperm |
| --- | --- | --- |
| 01 | 1 | 1 |
|  | 2 | 3 |
|  | 3 | 2 |
| 02 | 1 | 1 |
|  | 2 | 1 |
|  | 3 | 2 |
| 03 | 1 | 0 |
|  | 2 | 0 |
|  | 3 | 0 |

**Table S3. Kids born after natural mating of bucks carrying transfected testis**

| **Number of**  **Mating** | **Buck with**  **transfected testis** | **Doe** | **Kids born** |
| --- | --- | --- | --- |
| 1 | 01 | 001 | 1, 2 |
| 2 |  | 002 | 3, 4 |
| 3 |  | 003 | 5 |
| 4 |  | 004 | 6 |
| 5 |  | 005 | 7 |
| 6 |  | 006 | 8 |
| **5** | 02 | **007** | **9** |
| 6 |  | 008 | 10, 11, 12 |
| 9 | 03 | 009 | 13 |

Total 9 mating produced 13 kids. The mating (serial No. 5) which generated transgenic kid (Kid #9) is indicated as bold letters.

**Table S4. Weight of the testis used for *in vitro* transfection study. Statistical analysis was performed using Student's t-test (^**^*P*<0.01)**

| SI No | Age group (Year) | Weight of testis (g) | Average weight of testis ± s.e.m. (g) |
| --- | --- | --- | --- |
| 1 | 0.3-0.4 | 36.67 | 39.09±1.58 |
| 2 |  | 38.23 |  |
| 3 |  | 39.58 |  |
| 4 |  | 33.45 |  |
| 5 |  | 44.23 |  |
| 6 |  | 42.31 |  |
| 1 | ~3 | 69.45 | 75.43±1.82^**^ |
| 2 |  | 71.94 |  |
| 3 |  | 73.53 |  |
| 4 |  | 77.45 |  |
| 5 |  | 80.34 |  |
| 6 |  | 79.88 |  |

**Table S5. Plasmid concentrations used for optimization of *in vitro* electroporation of goat testes collected from the abattoir.** Three testes were used for each plasmid concentration.

| **SI No** | **Animal** | **Plasmid volume (ml)** | **Plasmid concentration (µg/µl)** |
| --- | --- | --- | --- |
| 1 | Pre-pubertal male | 1.0 | 0.10 |
|  |  |  | 0.25 |
|  |  |  | 0.50 |
|  |  |  | 1.00 |
|  |  |  | 1.50 |
| 2 | Adult male | 1.5 | 0.10 |
|  |  |  | 0.25 |
|  |  |  | 0.50 |
|  |  |  | 1.00 |
|  |  |  | 1.50 |

**Table S6. Gene-specific primers used for mRNA expression analysis**

| **SI No.** | **Gene** | **Primer sequence (5**′**-3**′**)** | **Product size (bp)** |
| --- | --- | --- | --- |
| **1** | *EGFP* | F: CCGACCACTACCAGCAGAACAC | 104 |
|  |  | R: CTCGTTGGGGTCTTTGCTCAG |  |
| **2** | *ACTB* | F: AGCTCGCCATGGATGATGA | 54 |
|  |  | R: TGCCGGAGCCGTTGT |  |

**Supplementary materials and methods**

**Isolation and culture of seminiferous tubules**

Immediately after electroporation, the testis was cut into small pieces. Then the pieces of interstitial tissues were minced with a pair of scissors. Seminiferous tubules were isolated by stripping off the adjacent connective tissue using forceps. The isolated seminiferous tubules were first transferred to a Petri plate containing Dulbecco’s Minimum Essential Medium (DMEM; Hyclone, USA) and then to 24 well plate (one plate/testis) containing the culture medium of DMEM and Ham’s F-12 (Gibco, USA) in 1:1 ratio containing 10% fetal bovine serum (FBS) and 1X antibiotic-antimycotic solution. The plates were incubated in a CO_2_ incubator at 37^0^C, 100% RH, and 5% CO_2_. The cultured cells were examined for fluorescence from d3 onwards. After the d14, the number of wells showing green fluorescence was counted and images were captured using a fluorescence microscope.

**Enzymatic isolation of testicular cells**

After removing the tunica albuginea and connective tissue, the electroporated testis was minced into small pieces on a petri dish containing DMEM and 1X antibiotic-antimycotic solution. The minced pieces were sequentially treated with 1 mg/ml collagenase IV (Gibco, USA) in DMEM for 30 min, 7 mg/ml DNase I in DMEM for 5 min and 0.25% trypsin-EDTA (Invitrogen, USA) in PBS for 20 min in a shaking water bath at 37°C. The cell suspension resulting from each stage was washed by centrifugation (800 rpm, 5 min) with pre-warmed (37°C) PBS. After washing, it was filtered through a 100 µm and 40 µm nylon cell strainers (BD Falcon, USA). The cell pellet was finally resuspended in DMEM containing 5% FBS. The number and viability of isolated testicular cells were determined by trypan blue staining.

***In vitro* culture of spermatogonial stem cells (SSCs)**

The differential plating technique was used to enrich SSCs from testicular cells. Briefly, to enrich spermatogonial stem cells, isolated testicular cells (~3X10^6^ cells per well) were suspended in DMEM/F12 with 10% FBS and 1X antibiotic–antimycotic solution for overnight in uncoated six-well plates. Then, suspended cells were collected, washed and co-cultured (1X10^5^ per well) on mitotically inactivated Sertoli cells in SSC medium at 37°C in a humidified incubator with 5% CO_2_. SSC medium consisted of DMEM-F12 supplemented with 10 % FBS, 15 ng/mL recombinant human GDNF (Sigma, USA), 1× essential amino acids (EAA; Gibco, USA), 1× Non-essential amino acids (NEAA; Gibco, USA) and 1X antibiotic–antimycotic solution. The culture medium was replaced on alternate days.

**Immunohistochemical (IHC) analysis**

After fixation in PFA, the testes samples were dehydrated in ascending ethanol series. Then the tissue samples were embedded in paraffin and sectioned at 5 μm using standard procedures. The sections were dewaxed, rehydrated and washed with 1X PBS/0.1% Tween-20 (PBST). Some slides, after mounted with Fluoroshield (Sigma, USA), were directly observed under a fluorescence microscope. Other slides were kept in antigen retrieval solution (0.5% trypsin and 1% CaCl_2_, pH 7.8) for 10 min. Then the slides were blocked in 5% BSA in PBS for 30 min at room temperature (RT). After blocking, the slides were incubated overnight at 4°C with the primary antibody, rabbit polyclonal anti-GFP (1:100; Sigma). The samples were washed three times with PBS and then incubated with the secondary antibody, HRP-conjugated chicken anti-rabbit GFP (1:500; Santa Cruz), for 1 h. After washing the slides with PBS, DAB solution was added to the smear and further incubated for 10 min at RT in the dark. The slides were counterstained with Haematoxylin for 2-3 min, and then washed under a running tap water and air dried. Finally, slides were mounted with DPX mountant (Sigma, USA) and examined under a phase contrast microscope.

**Reverse transcription PCR (RT-PCR) and Quantitative Real-Time PCR (qPCR) analysis**

The total RNA was extracted from the seminiferous tubules, semen and, blood and skin samples from kids using TRIzol reagent (Invitrogen, USA) following the manufacturer’s instructions. Total RNA from the fluorescent embryos was extracted using the RNAeasy micro kit (Qiagen, Netherlands). The isolated RNA was treated with DNA-free DNase kit (Ambion, USA). The concentration and purity of RNA were determined spectrophotometrically. The integrity of the RNA samples was ascertained using agarose gel electrophoresis. DNA-free RNA sample was used for reverse transcription in a final volume of 20 µl according to the manufacturer’s protocol (RevertAid H Minus First Strand cDNA Synthesis Kit, Fermentas, USA). Synthesized single-stranded cDNA was stored at -20°C until further use.

The expression of *EGFP* in the buck semen, testis of pre-pubertal goats, embryos, and blood and skin samples of baby goats generated from TMGT procedure were assessed using RT-PCR analysis. Quantitative real-time PCR was used to assess the expression of *EGFP* mRNA in the testicular samples of *in vivo* gene transferred pre-pubertal bucks and to detect the presence of *EGFP* gene in the semen from the three pre-founder bucks. β-actin (*ACTB*) was used as a reference gene for qPCR analysis. The gene-specific primer pairs for *EGFP* and *ACTB* (Supplementary Table S6) were designed using Primer Express (Ver 3.0). The qPCR was carried out using 7500 real-time PCR system (Applied Biosystems, USA). All PCR reactions were performed in triplicates in a reaction volume of 20 µl containing 1X Power SYBR® Green Master Mix (Applied Biosystems, USA), 50 nM of each gene-specific primer and 40 ng of cDNA template. PCR cycling conditions were as: 50°C for 2 min, initial denaturation at 95°C for 10 min, 40 cycles of 95°C for 15 sec 60°C for 60 sec.

**Western blotting**

Total protein was extracted from the seminiferous tubules using T-PER Reagent (Pierce, USA). A 25 µl of whole protein extract was separated by SDS-PAGE (12% resolving gel) and then transferred to a PVDF membrane (BiotraceTM PVDF, PALL Corporation, India) using a Semi-dry Electrophoretic Apparatus (Atto, Japan). The membrane was incubated with a blocking buffer (5% (w/v) skim milk powder and 0.25% (v/v) Tween-20 in PBS, pH 7.4) at 4°C for overnight. After blocking, the membrane was rocked gently with the primary antibody, rabbit polyclonal anti-GFP (1:500; Sigma, USA), for 2 h, followed by incubation with the secondary antibody, chicken anti-rabbit IgG-HRP (1:1000; Santa Cruz Biotechnology, USA), for 1 h. The detection was carried out in a dark room using ECL Western Blotting Substrate (Pierce, USA). The membrane was scanned using a G: BOX Imager (Syngene, England) for detection of the chemiluminescence.

***In vitro* fertilization (IVF)**

The method of *in vitro* production of embryos involved three main steps: *in vitro* maturation (IVM) of oocytes, *in vitro* fertilization (IVF) of oocytes and *in vitro* culture (IVC) of embryos.

***Oocyte collection and in vitro maturation***: Goat ovaries were collected from a local abattoir and transported to the laboratory in normal saline containing antibiotics (Penicillin 400 IU and Streptomycin 400 µg/ml) at 37^0^C. It was ensured that the ovaries are brought to the laboratory within 30 min of slaughter. The oocytes were collected from each ovarian follicle by aspiration using an 18-G needle containing oocyte collection medium (OCM) supplemented with 0.3% BSA and 50 µg/ml Gentamicin under a stereo-zoom microscope (Olympus, Japan). The oocytes were washed three times with IVM medium consisted of TCM199 (Sigma, USA) supplemented with 10% heat-inactivated FBS, 0.25 mM pyruvate, 50 µg/ml Gentamicin, 1 µg /ml 17β-estradiol, 0.5 µg /ml FSH and 100 µg/ml LH. Only A grade (>5 layer cumulus-oocyte complex; COC), B grade (3-5 layer COC) and C grade (<3 layer COC) oocytes were matured in 60 µl droplets of IVM medium in Petri dishes (35 mm X 10 mm) for 27h in a CO_2_ incubator (New Brunswick, Eppendorf, USA) maintained at 38.5^0^C, 5% CO_2_ and maximum humidity.

***Sperm preparation and in vitro fertilization***: Fresh semen (40 µl) was collected from bucks d45 post-electroporation using a artificial vagina. In the laboratory, semen was washed two times with Sperm-TALP (S-TALP) medium at 600 rpm for 5min. The sperm pellet was dissolved in 1.0 ml of Fertilization-TALP (F-TALP) medium containing 50 µg/ml heparin in S-TALP and incubated at 38.5°C in 5% CO_2_ incubator for 1 h. Meanwhile, the matured oocytes were washed in F-TALP and then pre-incubated at 38.5^0^C in a 40 µl of the F-TALP drop as 15-20 oocytes per drop. Finally, a 20 µl (2X10^6^ spermatozoa/ml) of capacitated sperm solution was added into each drop containing pre-incubated oocytes and the drops were incubated at 38.5^0^C, 5% CO_2_ in the air and maximum humidity for about 18 h.

***In vitro culture of embryos***: After 18h of co-incubation, fertilized oocytes were washed 4 to 5 times with modified Synthetic Oviductal Fluid (mSOF) medium. These oocytes were further cultured in mSOF in at 38.5^0^C, 5% CO_2_ in the air and maximum humidity. The medium was changed after 48 h of culture and embryos were examined for cleavage rate under an inverted microscope. Then the cleaved embryos were assessed under a fluorescence microscope for *EGFP* expression. Fluorescent embryos were pooled and the total RNA was extracted using RNeasy Mini Kit (Qiagen, Germany). In order to assess the mRNA expression of *EGFP* in the embryos, RT-PCR analysis was performed.

**PCR analysis**

The genomic DNA of kids produced from bucks carrying transfected testis was subjected to PCR analysis using transgene-specific primers. The plasmid, *pIRES2-EGFP* was used as a positive control and the genomic DNA of a wild (i.e., non-gene transferred) goat was used as a negative sample. The PCR primer pair designed to amplify a 617-bp fragment the *EGFP* sequence as follows: pEGFP1 FP, 5′-ACGTAAACGGCCACAAGTTC-3′ and pEGFP1 RP, 5′-GGCGGTCACGAACTCCAG-3′.

**Southern blotting analysis**

Ten micrograms of blood genomic DNA was digested with XhoI, purified by ethanol precipitation and then separated by electrophoresis on a 0.8% agarose gel. The DNA was then transferred onto a positively charged nylon membrane (Roche, Switzerland) using a Vacuum Blot (Biometra, Germany). The membrane was pre-hybridized for 30 min at 45^0^C and then hybridized for overnight at 45^0^C with the DIG labelled *pIRES2-EGFP* as a probe. The DIG labelling was carried out using DIG-High Prime DNA Labeling kit (Roche Applied Science, Germany). Washings were performed with DIG-High Prime DNA Labeling and Detection Starter Kit II (Roche Applied Science, Germany). After hybridization and stringent washings, the membrane was incubated for 30 min in the blocking solution and then incubated for 30 min in Anti-Digoxigenin-AP antibody solution (Roche Applied Science, Germany). The membrane was subsequently incubated for 10 min with 1.0 ml of CSPD ready-to-use solution (Roche Applied Science, Germany). The chemiluminescent signals were captured on X-ray and were photographed using a bright light X-ray viewer.

**Supplementary figures**


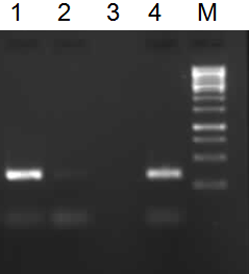


**Supplementary Figure 5C.** Actual gel picture of RT-PCR analysis reveals expression of *EGFP* mRNA in the fluorescent embryo produced using semen from *in vivo* gene transferred buck. 1; Positive control, 2 & 4; *EGFP* expressed embryo, 3; embryos obtained by IVF with wild sperm and M; 100 bp Marker.


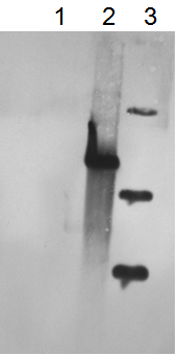


**Supplementary Figure 6C**. Southern blotting analysis showed the presence of the transgene in the kid produced from buck with transfected testis. 1: Genomic DNA from wild goat as a negative control, 2: Genomic DNA from the transgenic kid and 3: DNA marker.


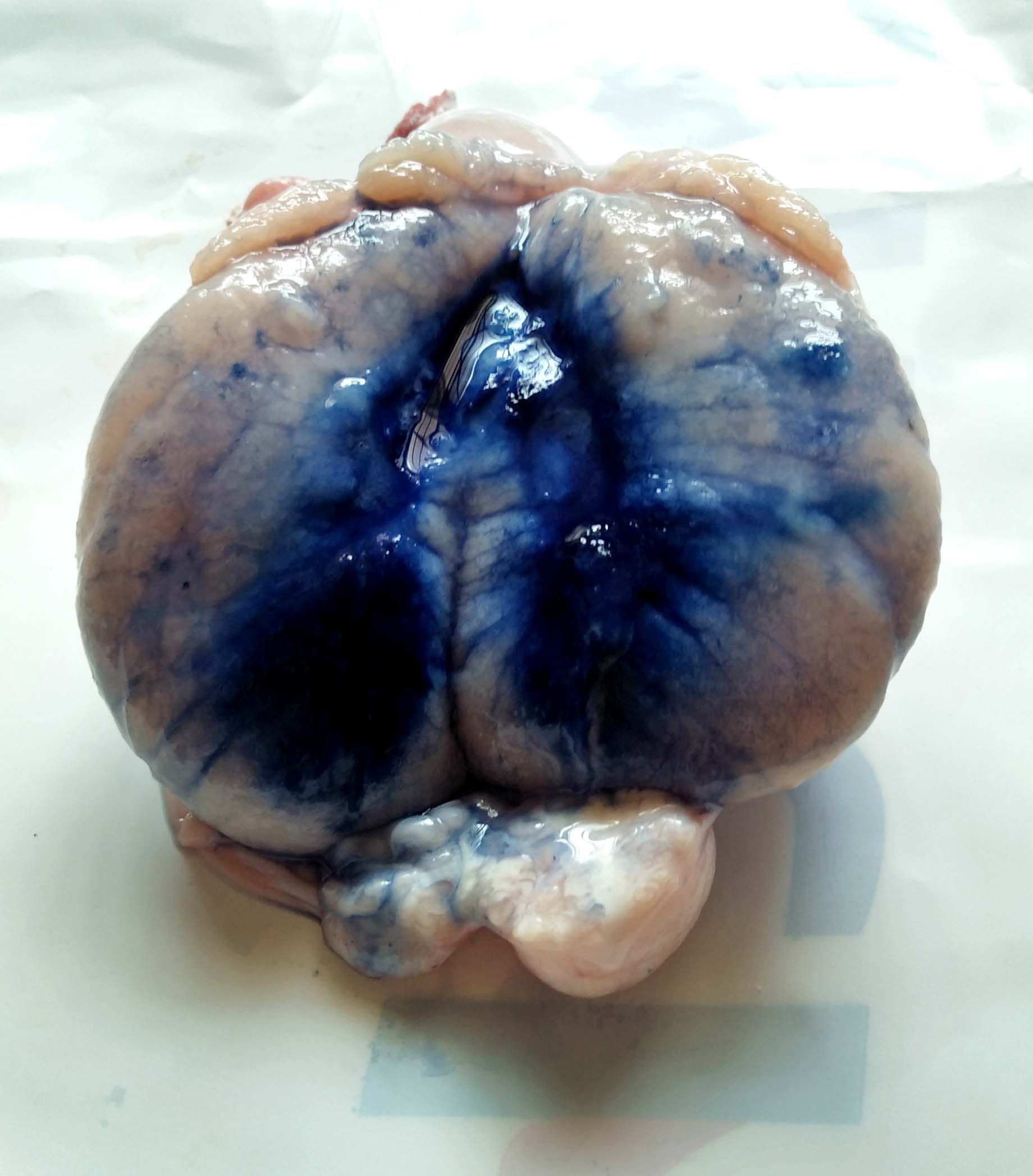


**Supplementary Figure S1:** Longitudinal section of adult goat testis showing a complete coverage of 1.5 ml of trypan blue aqueous solution. A 0.4% solution of trypan blue in buffered isotonic salt solution was injected *in vitro* to ten different sites of testicular interstitial space to confirm the optimum injection volume required for sufficient coverage of the testicular tissue.


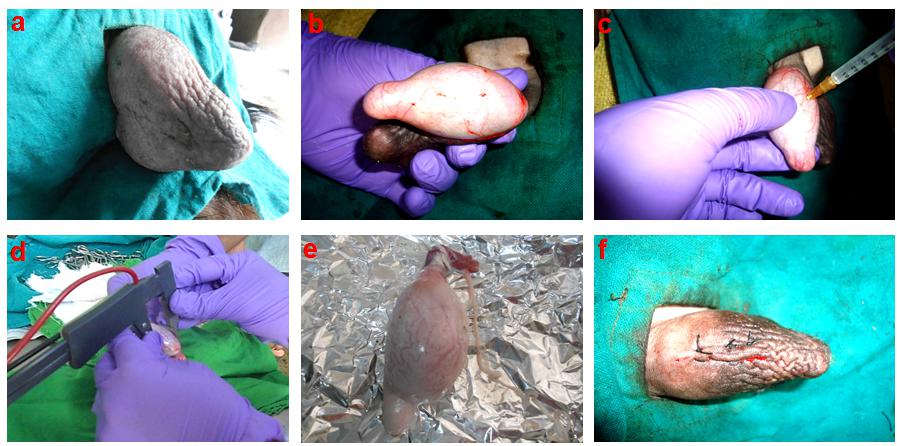


**Supplementary Figure S2**: *In vivo* electroporation of goat testis. **a:** Aseptically prepared scrotum, **b:** Testis was exposed in the scrotal sack and then fixed with the tip of fingers to avoid retraction during the injection, **c:** Linearized plasmid was injected into testicular interstitial space, **d:** Immediately after injection, testis was held between a pair of caliper-type electrodes (ECM830, Cat# 45-0102) and square electric pulses were applied using electric pulse generator (ECM830, BTX), **e:** Non-injected testis was removed by the separation of cremaster from the vascular testicular cord, **f:** Finally muscle and skin layers were sutured leaving a small gap for the exudation.
